# On simulations of 3D fractional WBBM model through mathematical and graphical analysis with the assists of fractionality and unrestricted parameters

**DOI:** 10.1038/s41598-024-61405-8

**Published:** 2024-07-16

**Authors:** Nur Hasan Mahmud Shahen, Foyjonnesa, Md. Al Amin, M. M. Rahman

**Affiliations:** 1 Department of Mathematics, Bangladesh University of Engineering and Technology, Dhaka, 1000 Bangladesh; 2 Department of Arts and Sciences, Bangladesh Army University of Science and Technology, Saidpur, 5310 Bangladesh; 3 Department of Mathematics, University of Rajshahi, Rajshahi, 6305 Bangladesh

**Keywords:** The 3D fractional WBBM equation family, The unified method, Water wave mechanics, Mathematical physics, Shallow water, Applied mathematics, Computational science, Computer science, Software

## Abstract

This study retrieves some novel exact solutions to the family of 3D space–time fractional Wazwaz–Benjamin–Bona–Mahony (WBBM) equations in the context of diverse nonlinear physical phenomena resulting from water wave mechanics. The family of WBBM equations is transformed for this purpose using a space and time fractional transformation into an ordinary differential equation (ODE). The ODE then uses a strong method, namely the Unified Method. Consequently, lump solutions, dark-bright soliton, singular and multiple soliton solutions, and periodic solutions are investigated. The disparities between the current study's conclusions and previously acquired solutions via other approaches are examined. All wave solutions produced are determined to be novel in terms of fractionality, unrestricted parameters, and implemented technique sense. The impact of unrestricted parameters and fractionality on the obtained solutions are visually presented, along with physical explanations. It is observed that the wave portents are varied with the increase of unrestricted parameters as well as fractionality. We dynamically showed that the appropriate transformation and the applied Unified approach more proficient in the study of water wave dynamics and might be used in future researches to clarify the many physical phenomena. The novelty of this work validate that the proposed method seem simple and useful tools for obtaining the solutions in PDEs and it is expected to use in mathematical physics and optical engineering.

## Introduction

A nonlinear partial differential equation (NPDE) is a mathematical equation that describes a relationship involving an unknown function and its partial derivatives, where the equation is not linear concerning the unknown function. The importance of NPDEs lies in their ability to model complex, real-world phenomena more accurately than linear partial differential equations (PDE). They are essential for describing chaotic systems, intricate pattern formation, turbulent fluid dynamics, and various physical and biological processes. NPDEs play a vital role in advancing our understanding and simulations in science, engineering, and applied mathematics.

Consider the following fractional version of the 3D WBBM equations^1,2^:

1 $${{\text{T}}}_{{\text{t}}}^{\upbeta }u+{{\text{T}}}_{{\text{x}}}^{\upbeta }u+{{\text{T}}}_{{\text{y}}}^{\upbeta }{u}^{3}-{{\text{T}}}_{{\text{xzt}}}^{3\upbeta }u=0,$$

2 $${{\text{T}}}_{{\text{t}}}^{\upbeta }u+{{\text{T}}}_{{\text{z}}}^{\upbeta }u+{{\text{T}}}_{{\text{x}}}^{\upbeta }{u}^{3}-{{\text{T}}}_{{\text{xyt}}}^{3\upbeta }u=0,$$

3 $${{\text{T}}}_{{\text{t}}}^{\upbeta }u+{{\text{T}}}_{{\text{x}}}^{\upbeta }u+{{\text{T}}}_{{\text{y}}}^{\upbeta }{u}^{3}-{{\text{T}}}_{{\text{xzt}}}^{3\upbeta }u=0.$$

In the given equations, $u(x,y,z,t)$ is a differentiable function involving four independent variables: $x, y, z,$ and $t.$ The notations ${T}_{x}^{\beta }u, {T}_{y}^{\beta }u, {T}_{z}^{\beta }u,$ and ${T}_{t}^{\beta }u$ represent the resultant derivatives of $u$ with regards to $x,y,z,$ and $t$, respectively and $0<\beta \le 1$ and $t\ge 0.$ Seadawy et al.^1^ elucidated these equations, exploring soliton solutions and their various characteristic.

Exact solutions to nonlinear PDEs are vital in understanding complex phenomena across diverse applied science fields. In recent times, with the emergence of advanced computational capabilities, there has been remarkable progress in addressing NPDEs through a diversity of significant mathematical and methodical approaches such as the novel ${\varphi }^{6}$ model expansion method^3^, the unified technique and the modified direct algebraic method^4^, and the modified extended $tanh$-function method^5,6^ play crucial roles. Alongside, the Simple Equation Technique^7^ and the Fan-extended sub equation approach^8^ contribute significantly. The exploration expands with the generalized exponential rational function strategy^9^ and the semi-inverse variational principle^10^, along with the sine–cosine method^11^, and the multiple $exp$-f unction system^12^. The modified ${\text{tan}}(\frac{\upphi \left(\upxi \right)}{2})$ and ${\text{tanh}}(\frac{\upphi \left(\upxi \right)}{2})$ methods^13,14^ and exp-function method^15^ bring additional insights. methods like the improved trial equation method^16^ and the extended rational trigonometric method^17^ further enrich the toolkit. The darboux transform method^18^, the exponential rational function method^19^, adomian decomposition method^20^, and the hirota’s bilinear method^21,22^ introduce innovative perspectives. The exp $(-\phi (\xi ))$-expansion^23^ and its extension methods^24–28^, along with the modified simple equation method^29^, and extended simple equation method^30^ offer versatile solutions. The investigation extends to specialized domains with methods like the extended $Sinh$-Gordon expansion method^31^ and the sine–Gordon expansion method^32^. In addition, the $(\frac{{G}^{\prime}}{{G}^{2}})$-expansion function method^33^, the Improved $(\frac{{G}^{\prime}}{{G}^{2}})$^34^, the extended Fan-sub equation method^35^, and $(\frac{{G}^{\prime}}{G},\frac{1}{G})$-expansion method^2^ provide a comprehensive understanding of nonlinear dynamics in diverse applications.

To solve the complex 3D WBBM equations^4,36^ different methods have been used. These methods include the Sine–Gordon expansion method^37^, the two-variable method^2^, the Improved $(\frac{{{\varvec{G}}}^{\boldsymbol{^{\prime}}}}{{{\varvec{G}}}^{2}})$^34^, the improved extended ${\varvec{t}}{\varvec{a}}{\varvec{n}}{\varvec{h}}$ function method^5^, the improved auxiliary equation technique^38^, etc.

Each of these approaches offers a unique way to understand and find solutions to the WBBM equations, adding to our knowledge of these mathematical models. When dealing with space–time fractional conformable equations, there isn't a one-size-fits-all method. Nonlinear equations are flexible, and tweaking a method can lead to discovering fresh solutions that are consistently beneficial. However, there are restrictions based on the specific challenges of prevailing methods and the familiarized approach. As the complexity of fractional differential equations increases (beyond four), finding solutions becomes tricky, and in some cases, there might be no solution. Researchers are therefore making great efforts to develop novel techniques and solutions for these kinds of equations.

The performance of long surface water waves in a two-dimensional ideal fluid is demonstrated by means of the space–time fractional 3D WBBM equation^34^. It is a generalization of the well-known KdV equation, and it has been used in research into the stability and solitonic behavior of water waves in a variety of physical systems^35^. The space–time fractional 3D WBBM equation has abundant applications in numerous fields of science. It is an essential tool for simulating long surface water waves in oceanography and fluid dynamics, offering insights into wave behavior in many aquatic environments^37^. The equation is used by environmental scientists to comprehend how waves affect coastal ecosystems and regions. Environmental scientists use this equation to understand how waves impact coastal regions and ecosystems. The equation permits the study of solitary wave solutions and their effects in many physical systems by generalising the KdV equation to the domain of solitons and nonlinear waves^38^. In mathematical physics, it is a helpful model to study the mathematical properties of NLEEs. Geophysicists can benefit from studying wave dynamics in geological environments, such as the propagation of tsunamis. The equation serves as a benchmark issue in numerical analysis and simulation, allowing researchers to assess the accuracy and efficiency of methods developed to solve NLEEs. Moreover, wave phenomena research in many other domains depends on the space–time fractional 3D WBBM equation.

In this study, we employ the Unified method^39,40^ to discover novel solutions for the WBBM equation. The method employed in this study for the WBBM equations is entirely novel. The resulting exact solutions not only provide valuable insights for further research in shallow water wave and mathematical physics but also serve as proof that our proposed WBBM equation, utilizing conformable derivatives, is well-suited for generating new traveling wave structures in various physical systems, free from the complications associated with obliqueness conditions. Because of its universal acceptance and appropriateness, the Unified approach has gained significant traction. It might have a relationship to several nonlinear equations and also provides two or three novel sorts of solutions^39^. Compared to other purported techniques^41–45^, our method is more efficient and reliable. The solutions derived by means of the previously described method can be characterized as hyperbolic, rational, and trigonometric functions. For reviewing particular nonlinear physical treatments, these types of solutions are suitable.

The novelty of this work validate that the proposed method seem simple and useful tools for obtaining the new soliton solutions in PDEs and it is expected to use in mathematical physics and water wave engineering. More explicitly the novelty and the contributions of this work are given in two aspects (i) The newly exact solitary solutions of 3D fractional WBBM equations are obtained by using the Unified method which are not obtained before (ii) Dynamical simulations of the proposed model through mathematical and graphical analysis with the assists of fractionality and unrestricted parameters. Moreover to the finest of our acquaintance, the received combined solitons have never been reported in other studies (Mamun et al.^2^; Shahen et al.^46^) in water wave mechanics.

The residue of this study is structured as follows, In Segment 2, we investigate into the meaning and various topographies of conformable derivatives (CD), along with the methods employed. "The unified technique application to the space–time fractional 3D WBBM equation family" section focuses on the application of this methodology to WBBM equations, providing insightful solutions. We present graphical depictions of the gained solutions in "Results and discussions". section. The last section summarizes our findings and provides a comprehensive conclusion.

## Groundworks and approaches

### Implication and some features of CD

Khalil et al.^47^ mainly established the concept of CD with the logic of limit.

#### Definition

If we consider a mapping $f:(0,\infty ) \to \Re \,,$ then the CD of $f$ order $\beta$ can be written as $T_{t}^{\beta } f(t) = \mathop {\lim }\limits_{\varepsilon \to 0} \left( {\frac{{f\,\left( {t + \varepsilon \,t^{1 - \beta } } \right) - f(t)}}{\varepsilon }} \right)\,,$ for all $t > 0,\;0 < \omega \le 1.$

Exponential functions rule, Gronwalls inequality, chain rule, definite as well as indefinite integration by slices, Fourier transformation, Laplace transform, moreover Taylor's power series developments for CD in the progression of fractional value of order have all been established in advance by one of the prominent researchers Abdeljawad^48^. The exertion of the current adapted Riemann Liouville derivative^48^ clarification can be smoothly overawed by the description of CD. Recently Shahen et al.^46^, Mamun et al.^6,34^, Rehman et al.^49^ and a group of scientist^50^, utilized this CD theory to solve some time fractional PDEs.

#### Theorem 1

Consider $\beta \in \,\,(0,\,1],$ and function $f = f(t),\,\,{\text{and }}g = g(t){\kern 1pt} ,$ be $\beta$-CD at the point $t > 0,$ thereafter we can state as

i. $$T_{t}^{\beta } \left( {cf + dg} \right) = cT_{t}^{\beta } f + dT_{t}^{\beta } g,$$ for all $$c,\,d \in \Re$$
ii. $T_{t}^{\beta } \left( {t * \gamma } \right) = \gamma \,t^{\gamma - \beta } ,\;$ for all $\gamma \in \Re$
iii. $$T_{t}^{\beta } \left( {f * g} \right) = gT_{t}^{\beta } \left( f \right) + f\;T_{t}^{\beta } \left( g \right)\,,\;$$
iv. $$T_{t}^{\beta } \left( \frac{f}{g} \right) = \frac{{gT_{t}^{\beta } \left( f \right) - f\,T_{t}^{\beta } \left( g \right)}}{{g^{2} }}.$$

Additionally, if this function $f$ is derivable, then $T_{t}^{\beta } \left( {f(t)} \right) = \,\,t^{1 - \beta } \frac{df}{{dt}}.$

#### Theorem 2

Take into consideration $f:(0,\beta ) \to R{\kern 1pt} {\kern 1pt} ,$ a function of real type such that $f$ is differentiable as well as $\beta$ conformable derivable. Additionally, let's assume that $g$ is a well-defined derivable function inside the range of $f$. After that, we have $T_{t}^{\beta } \left( {fog} \right)\,\left( t \right) = t^{1 - \beta } g(t)^{\beta - 1} \;g^{\prime}(t)\,T_{t}^{\beta } \left( {f(t)} \right)_{t = g(t)} \,,$ in where prime symbolizes the simple derivatives with relative to $\mathrm{the \; point} \; t$.

We were cautious in our study when it came to the preferred formula and the idea of derivatives based on conformability. Many functions are not enhanced Taylor's power order demonstrations on particular aspects in fundamental calculus, but they do consume the existence in the technique of CD. While intricate plans occur in the logic of fundamental fractional geometry, CD does well under the chain and product rules. Wherever the Riemann derivative of fractional order is not the topic, the CD of an unchanging kind of function corresponds to zero. Mittag Leffler functions appear a notable respected in fractional order of calculus as elucidation to exponential types of function in which the fractional order formula of exponential type functions of the form f(t) = e $\frac{{t}^{\alpha }}{\alpha },$ appears in context of CD.

The conformable derivative was chosen over alternatives such as local M derivative, Beta Derivative, etc., due to its efficacy in capturing the intricate dynamics of the space–time fractional 3D WBBM equation. Specifically, the conformable derivative ability to encapsulate local information and its applicability in describing the behavior of the soliton solutions (OSSs) within space–time fractional WBBM equation played a pivotal role in our method selection. We've elucidated on the advantages of the conformable derivative within the revised manuscript, highlighting its suitability for capturing the nuanced characteristics of the solutions derived using the Unified method.

### A concise overview of the unified technique

We will now delve into the Unified strategy for deriving precise solutions of a general NPDE in a step-by-step manner. Our investigation involves an NPDE that encompasses the independent variables $x, y, z,$ and $t$. The equation is presented below.

4 $$R\left(u, {{\text{T}}}_{{\text{t}}}^{\upbeta },{{\text{T}}}_{{\text{x}}}^{\upbeta },{{\text{T}}}_{{\text{y}}}^{\upbeta },{{\text{T}}}_{{\text{z}}}^{\upbeta },{{\text{T}}}_{{\text{xy}}}^{\mathrm{\beta \beta }},.\dots \dots \right)=0,$$

In the given context, $u\left(x,y,z,t\right)$ represents a function that is unknown, and $R$ characterizes a polynomial function involving both $u\left(x,y,z,t\right)$ as well as its partial derivative. This polynomial encompasses the nonlinear components as well as the derivative of the uppermost order.

Step-1: Here, we've looked at a traveling wave state that is stated as follows:
  5 $$u\left(x,y,z,t\right)=U\left(\xi \right) \; \text{and} \; \xi =l\frac{{x}^{\beta }}{\beta }+m\frac{{y}^{\beta }}{\beta }+n\frac{{z}^{\beta }}{\beta }-r\frac{{t}^{\beta }}{\beta },$$
  where $r$ represents a constant commonly referred to the velocity of the wave. To transform PDE into ODE, we substitute Eq. (5) in Eq. (4), resulting in the following equation.
  6 $$R\left(U,r{U}^{\prime},{lU}^{\prime},m{U}^{\prime},n{U}^{\prime},lm{U}^{{\prime}{\prime}},\dots \dots \right)=0,$$
Step-2: For the purpose of facilitate the integration of Eq. (6) multiple times, For the purpose of simplicity, we have expected that the integrating constant is zero.
Step-3: Regard the solution of the NLPDE in the following form:
  7 $$u\left(\xi \right)={A}_{0}+\sum_{i=1}^{N}\left[{A}_{i}{F}^{i}+{B}_{i}{F}^{-i}\right],$$
  where $F=F\left(\xi \right)$ of the form of Riccati differential equation
  8 $${F}^{\prime}\left(\xi \right)={F}^{2}\left(\xi \right)+b.$$
  where ${F}^{\prime}\left(\xi \right)=\frac{dF}{d\xi }, {A}_{i}, {B}_{i}$ and $b$ persist constants. Subsequently, general solutions of Eq. (8) are delivered as follows:
  **Family-1** For $b<0$, the responses of Eq. (8) take the hyperbolic-type function form:
  9 $$F\left(\xi \right)=\left\{\begin{array}{l}\frac{\sqrt{-b\left({A}^{2}+{B}^{2}\right)}-A\sqrt{-b}{\text{cosh}}\left(2\sqrt{-b}\left(\xi +C\right)\right)}{A{\text{sinh}}\left(2\sqrt{-b}\left(\xi +C\right)\right)+B}\\ \frac{-\sqrt{-b\left({A}^{2}+{B}^{2}\right)}-A\sqrt{-b}{\text{cosh}}\left(2\sqrt{-b}\left(\xi +C\right)\right)}{A{\text{sinh}}\left(2\sqrt{-b}\left(\xi +C\right)\right)+B}\\ \sqrt{-b}+\frac{-2A\sqrt{-b}}{A+{\text{cosh}}\left(2\sqrt{-b}\left(\xi +C\right)\right)-{\text{sinh}}\left(2\sqrt{-b}\left(\xi +C\right)\right)}\\ -\sqrt{-b}+\frac{2A\sqrt{-b}}{A+{\text{cosh}}\left(2\sqrt{-b}\left(\xi +C\right)\right)+{\text{sinh}}\left(2\sqrt{-b}\left(\xi +C\right)\right)},\end{array}\right.$$
  where $A$ and $B$ represent arbitrary constants as well as $C$ is another arbitrary constant.
  **Family-2** For $b>0$, the responses of Eq. (8) manifest in the form of trigonometric functions and are expressed as:
  10 $$F\left(\xi \right)=\left\{\begin{array}{l}\frac{\sqrt{b\left({A}^{2}-{B}^{2}\right)}-A\sqrt{b}{\text{cos}}\left(2\sqrt{b}\left(\xi +C\right)\right)}{A{\text{sin}}\left(2\sqrt{d}\left(\xi +C\right)\right)+B}\\ \frac{-\sqrt{b\left({A}^{2}-{B}^{2}\right)}-A\sqrt{b}{\text{cos}}\left(2\sqrt{b}\left(\xi +C\right)\right)}{A{\text{sin}}\left(2\sqrt{b}\left(\xi +C\right)\right)+B}\\ i\sqrt{b}+\frac{-2Ai\sqrt{b}}{A+{\text{cos}}\left(2\sqrt{b}\left(\xi +C\right)\right)-i{\text{sin}}\left(2\sqrt{b}\left(\xi +C\right)\right)}\\ -i\sqrt{b}+\frac{2Ai\sqrt{d}}{A+{\text{cos}}\left(2\sqrt{b}\left(\xi +C\right)\right)+i{\text{sin}}\left(2\sqrt{b}\left(\xi +C\right)\right)}.\end{array}\right.$$
  In this scenario, $A$ and $B$ denote arbitrary constants as well as $C$ is also considered an arbitrary constant.
  **Family-3** For $b=0,$ the responses of Eq. (8) take the form of rational functions:
  11 $$F\left(\xi \right)=-\frac{1}{\left(\xi +C\right)},$$
  where $C$ stands as a constant with arbitrary characteristics.
  **Family-4** In this step, N is a positive term that can be established by assuming the homogeneous equilibrium between the order of maximum derivatives and the highest order of nonlinear dispersive expressions as indicated in Eq. (6). If the degree of $u\left(\xi \right)$ as $D[u\left(\xi \right)]=N$, thereafter the degree of the term is specified by
  $$D\left[\frac{{d}^{q}u}{d{\xi }^{q}}\right]=N+q,$$
  $$D\left[{u}^{r}{\left(\frac{{d}^{q}u}{d{\xi }^{q}}\right)}^{s}\right]=Nr+s\left(q+N\right).$$
Step-5: By substituting Eq. (7) as well as Eq. (8) into Eq. (6), and grouping all terms with the similar degree of $F$, setting each coefficient of ${F}^{i}(-N\le i\le N)$ equivalent to zero, we acquire a family of algebraic equation (SAE).
Step-6: By substituting ${A}_{i}, {B}_{i}$, and $b$ in Eq. (7), which is discovered by SAE solving in step-5, and incorporating the general solutions of Eq. (8) into Eqs. (9), (10), as well as (11), It follows that the value of $b$ directly affects the explicit answers to Eq. (4).

## The unified technique application to the space–time fractional 3D WBBM equation family

Within this segment, we employ the method outlined in "A concise overview of the Unified technique" section to explore novel exact solutions concerning the fractional form of the WBBM^34^ equation family.

### The solutions of Eq. (1)

To address our specified equation, we adopt a wave transformation approach as $u\left(x,y,z,t\right)=U\left(\xi \right),$ where $\xi =l\frac{{x}^{\beta }}{\beta }+m\frac{{y}^{\beta }}{\beta }+n\frac{{z}^{\beta }}{\beta }-r\frac{{t}^{\beta }}{\beta }$, $r\ne 0$ and after switching out this transformation into Eq. (1), we derive the subsequent equation:

12 $$-r{u}^{\prime}+lu^{\prime}+m({u}^{3})^{\prime}+lnru^{{\prime}{\prime}{\prime}}=0.$$

By integrating Eq. (12) with respect to $\xi$ once and the integrating constant is set to zero, the resulting equation is obtained after interpretation as follows

13 $$\left(l-r\right)u+m{u}^{3}+lnr{u}^{{\prime}{\prime}}=0.$$

Following the concept of homogenous balance in Eq. (13), we conclude the value of $N=1$. Consequently, Eq. (7) is expressed as follows

14 $$U\left(\xi \right)={A}_{0}+{A}_{1}F\left(\xi \right)+{B}_{1}{F\left(\xi \right)}^{-1},$$

where ${A}_{0},{A}_{1}, and {B}_{1}$ represent real parameters that are presently unknown and will be determined later. Using Eqs. (8), (9), and (14) in Eq. (12), we obtained a polynomial in terms of $F(\xi )$. By putting all coefficients of $F(\xi )$) to zero, we obtain the SAE as follows:

$$2lnr{A}_{1}+m{A}_{1}^{3}=0$$

$$3m{A}_{0}{A}_{1}^{2}=0$$

$$2blnr{A}_{1}+3m{A}_{0}^{2}{A}_{1}+3m{A}_{1}^{2}{B}_{1}+l{A}_{1}-r{A}_{1}=0$$

$$m{A}_{0}^{3}+6m{A}_{0}{A}_{1}{B}_{1}+l{A}_{0}-r{A}_{0}=0$$

$$2blnr{B}_{1}+3m{A}_{0}^{2}{B}_{1}+3m{A}_{1}{B}_{1}^{2}+l{B}_{1}-r{B}_{1}=0$$

$$3m{A}_{0}{B}_{1}^{2}=0$$

$$2{b}^{2}lnr{B}_{1}+m{B}_{1}^{3}=0.$$

Solving these SAE, we get 2 sets of solutions. From the solutions, we take one solution for further investigation, and the solution is as follows:

15 $$r= -\frac{l}{8bln-1},{A}_{0}=0,{A}_{1}=\pm \frac{\sqrt{2}\sqrt{m\left(8bln-1\right)n}}{m\left(8bln-1\right)}l,{B}_{1}=\mp \frac{\sqrt{2}\sqrt{m\left(8bln-1\right)n}}{m\left(8bln-1\right)}lb.$$

**Family 1** (When $$b<0$$)

By utilizing the solution of the SAE and Eq. (9), we gain the following general traveling wave-solutions for the Eq. (14).

$${U}_{\mathrm{1,2},\mathrm{3,4}}\left(x,y,z,t\right)= \pm \frac{\sqrt{2}\sqrt{\alpha n}l\rho }{\alpha \eta }\mp \frac{\sqrt{2}\sqrt{\alpha n}lb\eta }{\alpha \rho }$$

$${U}_{\mathrm{5,6}}\left(x,y,z,t\right)=\pm \frac{1}{\alpha }\left(\sqrt{2} \sqrt{\alpha n}l\right)\mu \mp \frac{\sqrt{2}\sqrt{\alpha n}lb}{\alpha \mu }$$

$${U}_{\mathrm{7,8}}\left(x,y,z,t\right)=\pm \frac{1}{\alpha }\left(\sqrt{2} \sqrt{\alpha n}l\right)\varphi \mp \frac{\sqrt{2}\sqrt{\alpha n}lb}{\alpha \varphi },$$

where $$\alpha = m\left(8bln-1\right),$$ $$\rho = \sqrt{-\left({A}^{2}+{B}^{2}\right)b}-A\sqrt{-b}{\text{cosh}}\left(2\sqrt{-b}\left(\xi +C\right)\right),$$ $$\eta =A{\text{sinh}}\left(2\sqrt{-b}\left(\xi +C\right)\right)+B,$$ $$\mu =\sqrt{-b}-\frac{2A\sqrt{-b}}{A+{\text{cosh}}\left(2\sqrt{-b} \left(\xi +C\right)-{\text{sinh}}(2\sqrt{-b} \left(\xi +C\right)\right)}$$ and $$\varphi =-\sqrt{-b}+\frac{2A\sqrt{-b}}{A+{\text{cosh}}\left(2\sqrt{-b} \left(\xi +C\right)+{\text{sinh}}(2\sqrt{-b} \left(\xi +C\right)\right)}$$.

**Family 2** (When $$b>0$$)

By utilizing the solution of the SAE and Eq. (10), we gain the following general itinerant wave solutions for the Eq. (14).

$${U}_{\mathrm{9,10,11,12}}\left(x,y,z,t\right)= \pm \frac{\sqrt{2}\sqrt{\alpha n}l\tau }{\alpha \sigma }\mp \frac{\sqrt{2}\sqrt{\alpha n}lb\sigma }{\alpha \tau }$$

$${U}_{\mathrm{13,14}}\left(x,y,z,t\right)=\pm \frac{1}{\alpha }\left(\sqrt{2} \sqrt{\alpha n}l\right)\nu \mp \frac{\sqrt{2}\sqrt{\alpha n}lb}{\alpha \nu }$$

$${U}_{\mathrm{15,16}}\left(x,y,z,t\right)=\pm \frac{1}{\alpha }\left(\sqrt{2} \sqrt{\alpha n}l\right)\omega \mp \frac{\sqrt{2}\sqrt{\alpha n}lb}{\alpha \omega },$$

where $$\alpha = m\left(8bln-1\right),$$ $$\tau = \sqrt{\left({A}^{2}+{B}^{2}\right)b}-A\sqrt{b}{\text{cos}}\left(2\sqrt{b}\left(\xi +C\right)\right),$$

$$\nu =i\sqrt{b}-\frac{2iA\sqrt{b}}{A+{\text{cos}}\left(2\sqrt{b} \left(\xi +C\right)-i{\text{sin}}(2\sqrt{b} \left(\xi +C\right)\right)},$$ $$\sigma = A{\text{sin}}\left(2\sqrt{b}\left(\xi +C\right)\right)+B,$$ $$\omega =-i\sqrt{b}+\frac{2iA\sqrt{b}}{A+{\text{cos}}\left(2\sqrt{b} \left(\xi +C\right)+{\text{sin}}(2\sqrt{b} \left(\xi +C\right)\right)},$$ and $$i=\sqrt{-1}.$$

**Family 3** (When $$b=0$$)

By utilizing the solution of the SAE and Eq. (11), we gain the subsequent general itinerant wave solutions for the Eq. (14).

$${U}_{\mathrm{17,18}}\left(x,y,z,t\right)=\pm \frac{\sqrt{2}\sqrt{-mn}l}{m(\xi +C)}.$$

### The solutions of Eq. (2)

Again, we consider the same wave transformation $u\left(x,y,z,t\right)=U\left(\xi \right),$ where $\xi =l\frac{{x}^{\beta }}{\beta }+m\frac{{y}^{\beta }}{\beta }+n\frac{{z}^{\beta }}{\beta }-r\frac{{t}^{\beta }}{\beta }$, $r\ne 0$ and after switching out this transformation into Eq. (2), the resulting equation is as follows

16 $$-r{u}^{\prime}+n{u}^{\prime}+l{\left({u}^{3}\right)}^{\prime}+lmr{u}^{{\prime}{\prime}{\prime}}=0.$$

By integrating Eq. (16) with regard to $\xi$ once and the integrating constant is set to zero, the resulting equation is obtained after interpretation

17 $$\left(n-r\right)u+l{u}^{3}+lmr{u}^{{\prime}{\prime}}=0.$$

After plugging the homogeneous equilibrium principle in Eq. (17), we obtaining the assessment of $N=1.$ Then Eq. (7) takes the following form:

18 $$U\left(\xi \right)={A}_{0}+{A}_{1}F\left(\xi \right)+{B}_{1}{F\left(\xi \right)}^{-1},$$

where ${A}_{0}, {A}_{1} and {B}_{1}$ represent real parameters that are presently unknown and will be determined later. By substituting Eqs. (8), (9), as well as (18) into Eq. (17), we obtain the same type of SAE as before. Solving these equations yields some solutions one of which is as follows:

19 $$r= -\frac{n}{8blm-1},{A}_{0}=0, {A}_{1}=\pm \frac{\sqrt{2}\sqrt{m\left(8blm-1\right)nm}}{\left(8blm-1\right)},{B}_{1}=\mp \frac{\sqrt{2}\sqrt{m\left(8blm-1\right)nm}}{\left(8bln-1\right)}b.$$

**Family 1** (When b < 0)

By applying the solution of the SAE and Eq. (9) to Eq. (18), we attain the following general traveling wave solutions.

$${U}_{\mathrm{19,20,21,22}}\left(x,y,z,t\right)= \pm \frac{\sqrt{2}\sqrt{\alpha nm}\rho }{\alpha \eta }\mp \frac{\sqrt{2}\sqrt{\alpha nm}b\eta }{\alpha \rho }$$

$${U}_{\mathrm{23,24}}\left(x,y,z,t\right)=\pm \frac{1}{\alpha }\left(\sqrt{2} \sqrt{\alpha nm}\right)\mu \mp \frac{\sqrt{2}\sqrt{\alpha nm}b}{\alpha \mu }$$

$${U}_{\mathrm{25,26}}\left(x,y,z,t\right)=\pm \frac{1}{\alpha }\left(\sqrt{2} \sqrt{\alpha nm}\right)\varphi \mp \frac{\sqrt{2}\sqrt{\alpha nm}b}{\alpha \varphi },$$

where $$\alpha = \left(8blm-1\right),$$ $$\rho = \sqrt{-\left({A}^{2}+{B}^{2}\right)b}-A\sqrt{-b}{\text{cosh}}\left(2\sqrt{-b}\left(\xi +C\right)\right),$$ $$\eta =A{\text{sinh}}\left(2\sqrt{-b}\left(\xi +C\right)\right)+B,$$ $$\mu =\sqrt{-b}-\frac{2A\sqrt{-b}}{A+{\text{cosh}}\left(2\sqrt{-b} \left(\xi +C\right)-{\text{sinh}}(2\sqrt{-b} \left(\xi +C\right)\right)}$$ and $$\varphi =-\sqrt{-b}+\frac{2A\sqrt{-b}}{A+{\text{cosh}}\left(2\sqrt{-b} \left(\xi +C\right)+{\text{sinh}}(2\sqrt{-b} \left(\xi +C\right)\right)}$$.

**Family 2** (When b > 0)

By applying the solution of the SAE and Eq. (10) to Eq. (18), we attain the following general traveling wave solutions.

$${U}_{\mathrm{27,28,29,30}}\left(x,y,z,t\right)= \pm \frac{\sqrt{2}\sqrt{\alpha nm}\tau }{\alpha \sigma }\mp \frac{\sqrt{2}\sqrt{\alpha nm}b\sigma }{\alpha \tau }$$

$${U}_{\mathrm{31,32}}\left(x,y,z,t\right)=\pm \frac{1}{\alpha }\left(\sqrt{2} \sqrt{\alpha nm}\right)\nu \mp \frac{\sqrt{2}\sqrt{\alpha nm}b}{\alpha \nu }$$

$${U}_{\mathrm{33,34}}\left(x,y,z,t\right)=\pm \frac{1}{\alpha }\left(\sqrt{2} \sqrt{\alpha nm}\right)\omega \mp \frac{\sqrt{2}\sqrt{\alpha nm}b}{\alpha \omega },$$

where $$\alpha = \left(8blm-1\right),$$ $$\tau = \sqrt{\left({A}^{2}+{B}^{2}\right)b}-A\sqrt{b}{\text{cos}}\left(2\sqrt{b}\left(\xi +C\right)\right),$$

$$\nu =i\sqrt{b}-\frac{2iA\sqrt{b}}{A+{\text{cos}}\left(2\sqrt{b} \left(\xi +C\right)-i{\text{sin}}(2\sqrt{b} \left(\xi +C\right)\right)},$$ $$\sigma = A{\text{sin}}\left(2\sqrt{b}\left(\xi +C\right)\right)+B,$$ $$\omega =-i\sqrt{b}+\frac{2iA\sqrt{b}}{A+{\text{cos}}\left(2\sqrt{b} \left(\xi +C\right)+{\text{sin}}(2\sqrt{b} \left(\xi +C\right)\right)},$$ and $$i=\sqrt{-1}.$$

**Family 3** (When $$b=0$$)

By applying the solution of the SAE and Eq. (11) to Eq. (18), we obtain the following general traveling wave solution:

$${U}_{\mathrm{35,36}}\left(x,y,z,t\right)=\pm \frac{\sqrt{2}\sqrt{-mn}}{(\xi +C)}.$$

### The solutions of Eq. (3)

By using the same wave transformation i.e., $u\left(x,y,z,t\right)=U\left(\xi \right),$ where $\xi =l\frac{{x}^{\beta }}{\beta }+m\frac{{y}^{\beta }}{\beta }+n\frac{{z}^{\beta }}{\beta }-r\frac{{t}^{\beta }}{\beta }$, $r\ne 0$ and after switching out this transformation into Eq. (3), we obtain the succeeding equation

20 $$-r{u}^{\prime}+m{u}^{\prime}+n{\left({u}^{3}\right)}^{\prime}+{l}^{2}ru^{{\prime}{\prime}{\prime}}=0.$$

Integrating Eq. (20) with regard to $\xi$ for once and the integrating constant is set to zero, the resulting equation is obtained after simplification

21 $$\left(m-r\right)u+n{u}^{3}+{l}^{2}r{u}^{{\prime}{\prime}}=0.$$

After plugging the homogeneous equilibrium principle in Eq. (21), we determine the assessment of $N=1.$ Then Eq. (7) takes the following form:

22 $$U\left(\xi \right)={A}_{0}+{A}_{1}F\left(\xi \right)+{B}_{1}{F\left(\xi \right)}^{-1},$$

where ${A}_{0}, {A}_{1} and {B}_{1}$ are parameters of real type that are unknowns and resolved later. By substituting Eqs. (8), (9), and (22) into Eq. (21), we obtain the same type of SAE as before. Solving these equations yields some solutions one of which is as follows:

23 $$r= -\frac{m}{8b{l}^{2}-1},\; {A}_{0}=0, \; {A}_{1}=\pm \frac{\sqrt{2}\sqrt{n\left(8b{l}^{2}-1\right)m}}{n\left(8b{l}^{2}-1\right)}l, \; {B}_{1}=\mp \frac{\sqrt{2}\sqrt{m\left(8b{l}^{2}-1\right)n}}{n\left(8b{l}^{2}-1\right)}lb.$$

**Family 1** (When b < 0)

By employing the solution of the SAE Eq. (23) as well as Eq. (9) into Eq. (22), we find the following general traveling wave solution:

$${U}_{\mathrm{37,38,39,40}}\left(x,y,z,t\right)= \pm \frac{\sqrt{2}\sqrt{\alpha m}l\rho }{\alpha \eta }\mp \frac{\sqrt{2}\sqrt{\alpha m}lb\eta }{\alpha \rho }$$

$${U}_{\mathrm{41,42}}\left(x,y,z,t\right)=\pm \frac{1}{\alpha }\left(\sqrt{2} \sqrt{\alpha m}l\right)\mu \mp \frac{\sqrt{2}\sqrt{\alpha m}lb}{\alpha \mu }$$

$${U}_{\mathrm{43,44}}\left(x,y,z,t\right)=\pm \frac{1}{\alpha }\left(\sqrt{2} \sqrt{\alpha m}l\right)\varphi \mp \frac{\sqrt{2}\sqrt{\alpha m}lb}{\alpha \varphi },$$

where $$\alpha = n\left(8b{l}^{2}-1\right),$$ $$\rho = \sqrt{-\left({A}^{2}+{B}^{2}\right)b}-A\sqrt{-b}{\text{cosh}}\left(2\sqrt{-b}\left(\xi +C\right)\right),$$ $$\eta =A{\text{sinh}}\left(2\sqrt{-b}\left(\xi +C\right)\right)+B,$$ $$\mu =\sqrt{-b}-\frac{2A\sqrt{-b}}{A+{\text{cosh}}\left(2\sqrt{-b} \left(\xi +C\right)-{\text{sinh}}(2\sqrt{-b} \left(\xi +C\right)\right)}$$ and $$\varphi =-\sqrt{-b}+\frac{2A\sqrt{-b}}{A+{\text{cosh}}\left(2\sqrt{-b} \left(\xi +C\right)+{\text{sinh}}(2\sqrt{-b} \left(\xi +C\right)\right)}$$.

**Family 2** (When b > 0)

By employing the solution of the SAE and Eq. (10) in Eq. (22), we gain the subsequent general traveling wave solutions.

$${U}_{\mathrm{45,46,47,48}}\left(x,y,z,t\right)= \pm \frac{\sqrt{2}\sqrt{\alpha m}l\tau }{\alpha \sigma }\mp \frac{\sqrt{2}\sqrt{\alpha m}lb\sigma }{\alpha \tau }$$

$${U}_{\mathrm{49,50}}\left(x,y,z,t\right)=\pm \frac{1}{\alpha }\left(\sqrt{2} \sqrt{\alpha m}l\right)\nu \mp \frac{\sqrt{2}\sqrt{\alpha m}lb}{\alpha \nu }$$

$${U}_{\mathrm{51,52}}\left(x,y,z,t\right)=\pm \frac{1}{\alpha }\left(\sqrt{2} \sqrt{\alpha m}l\right)\omega \mp \frac{\sqrt{2}\sqrt{\alpha m}lb}{\alpha \omega },$$

where $$\alpha = n\left(8b{l}^{2}-1\right),$$ $$\tau = \sqrt{\left({A}^{2}+{B}^{2}\right)b}-A\sqrt{b}{\text{cos}}\left(2\sqrt{b}\left(\xi +C\right)\right),$$

$$\nu =i\sqrt{b}-\frac{2iA\sqrt{b}}{A+{\text{cos}}\left(2\sqrt{b} \left(\xi +C\right)-i{\text{sin}}(2\sqrt{b} \left(\xi +C\right)\right)},$$ $$\sigma = A{\text{sin}}\left(2\sqrt{b}\left(\xi +C\right)\right)+B,$$ $$\omega =-i\sqrt{b}+\frac{2iA\sqrt{b}}{A+{\text{cos}}\left(2\sqrt{b} \left(\xi +C\right)+{\text{sin}}(2\sqrt{b} \left(\xi +C\right)\right)},$$ and $$i=\sqrt{-1}.$$

**Family 3** (When $$b=0$$)

By employing the solution of the SAE as well as Eq. (11) in Eq. (22), we obtain the following general traveling wave solution:

$${U}_{\mathrm{53,54}}\left(x,y,z,t\right)=\pm \frac{\sqrt{2}\sqrt{-mn}l}{n(\xi +C)}.$$

## Results and discussions

The physical characterization of the founded precise solutions to the family of equations for 3D fractional WBBM will be covered in this canto. We give a graphical depiction of these solutions and discuss the various types of solutions. We have utilized two computational programs Maple-17 and MATLab to solve the equations and draw the graphs.

### Physical representation

**Figure 1:**
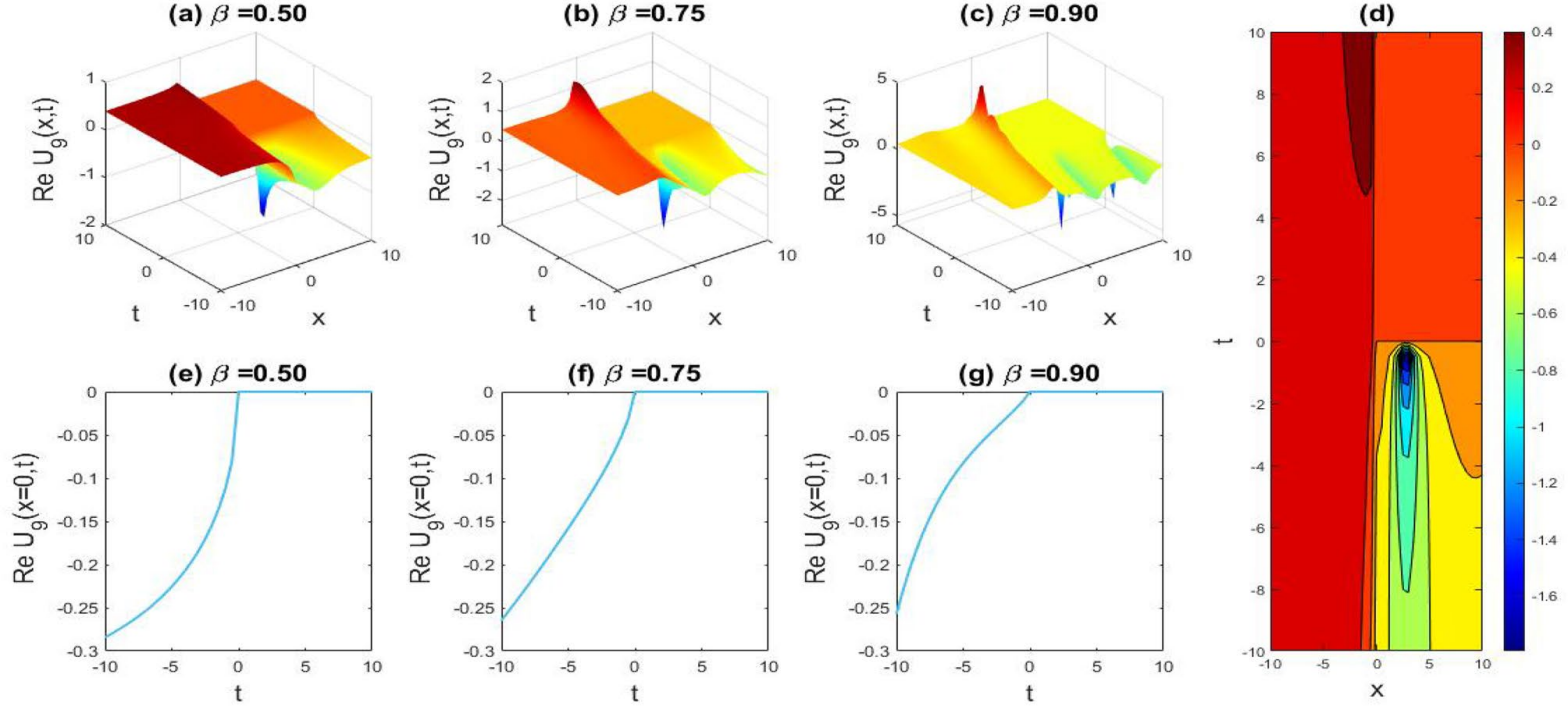
Illustrating the dynamic behavior derived from the solution of ${U}_{9}(x,y,z,t)$ with parameter values ${\text{A}}=-1,\mathrm{ B}=-1,\mathrm{ C}=-1,\mathrm{ b}=0.25,\mathrm{ l}=-0.2,\mathrm{ m}=1,\mathrm{ n}=1.$ Subfigures (**a**–**c**) provide 3D plot views, while (**d**) showcases the contour plot for $\beta =0.5$. Additionally, subfigures (**e**–**g**) feature 2D line graphs for $x=0$ corresponding to the respective figures (**a**–**c**).

**Figure 2:**
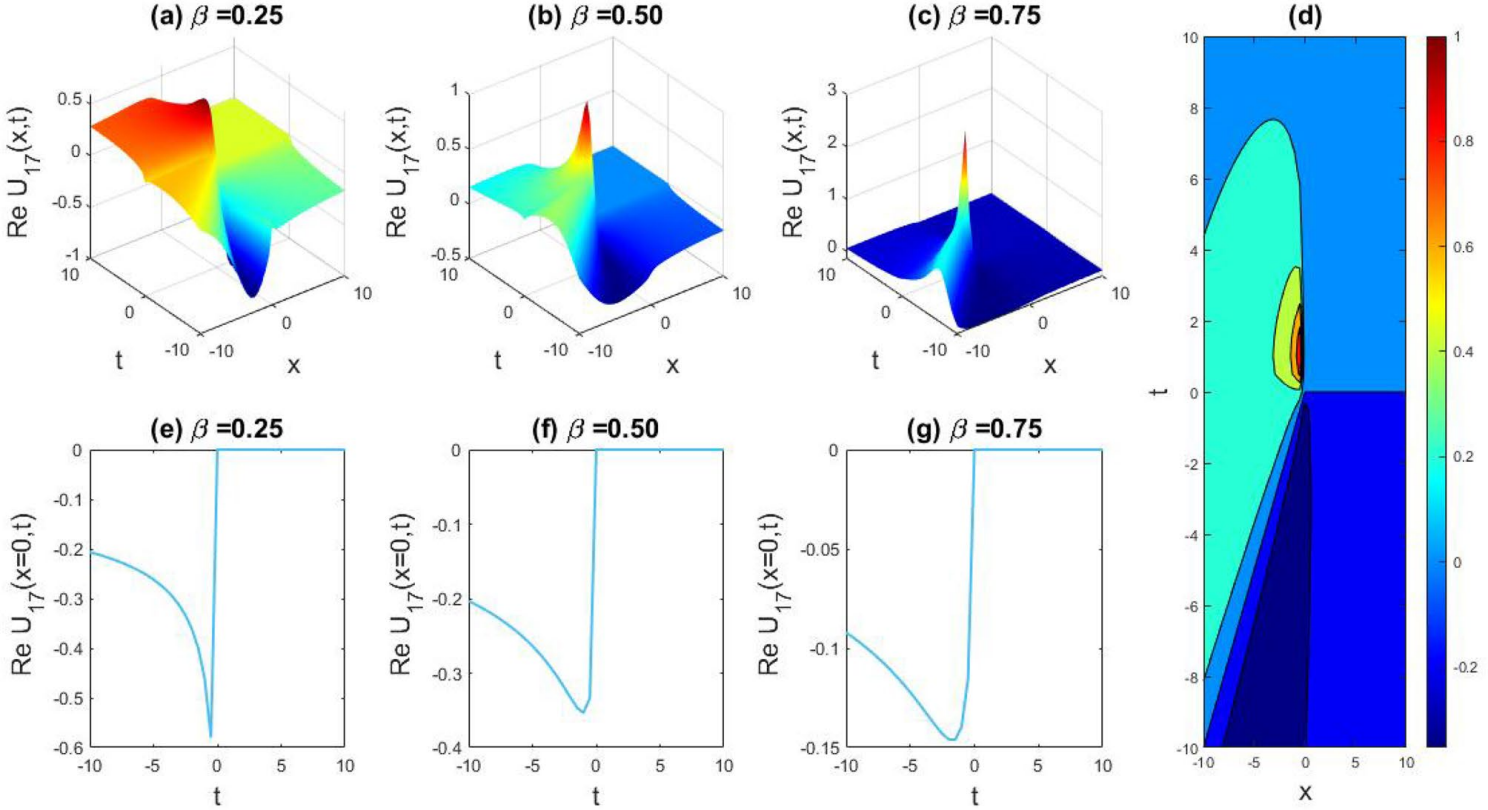
Capturing the dynamical evolution of ${U}_{17}(x, y, z, t$) with parameter values $A=-1, B=-1, C=-1, b=0, l=-0.5, m=1, n=1.$ Subfigures (**a**–**c**) showcase 3D plot views, (**d**) reveal the contour plot for $\beta =0.5,$ and subfigures (**e**–**g**) portray 2D line graphs for $x=0$ corresponding to (**a**–**c**) respectively.

**Figure 3:**
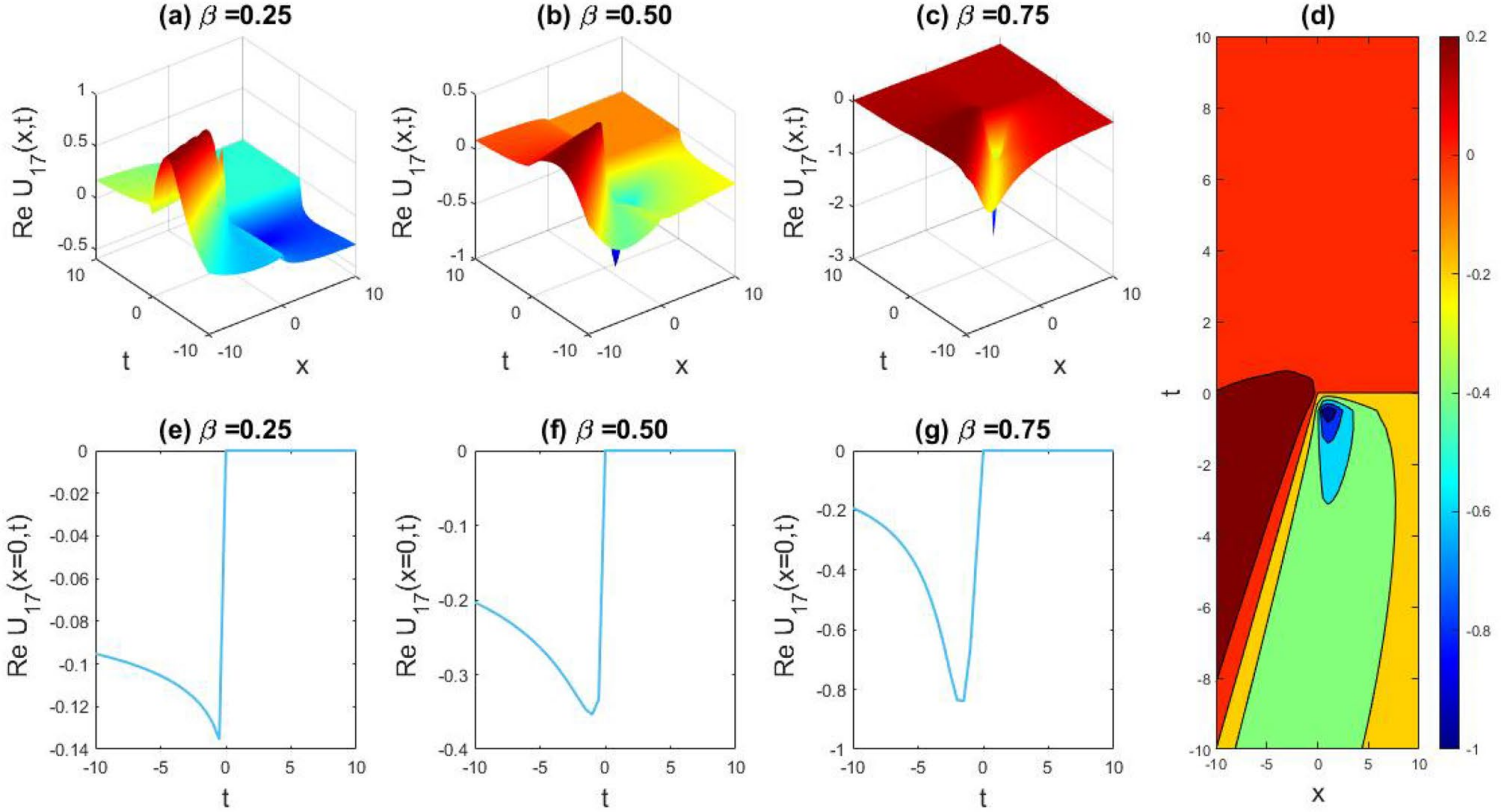
Capturing the dynamic behavior emerging from the solution of ${U}_{17}(x, y, z, t)$ with parameter values $A=-1, B=-1, C=-1, b=0, l=0.5, m=1, n=1.$ Subfigures (**a**–**c**) showcase 3D plot views, (**d**) features the contour plot for $\beta =0.5,$ and subfigures (**e**–**g**) illustrate 2D line graphs for $x=0$ corresponding to figures (**a**–**c**) respectively.

**Figure 4:**
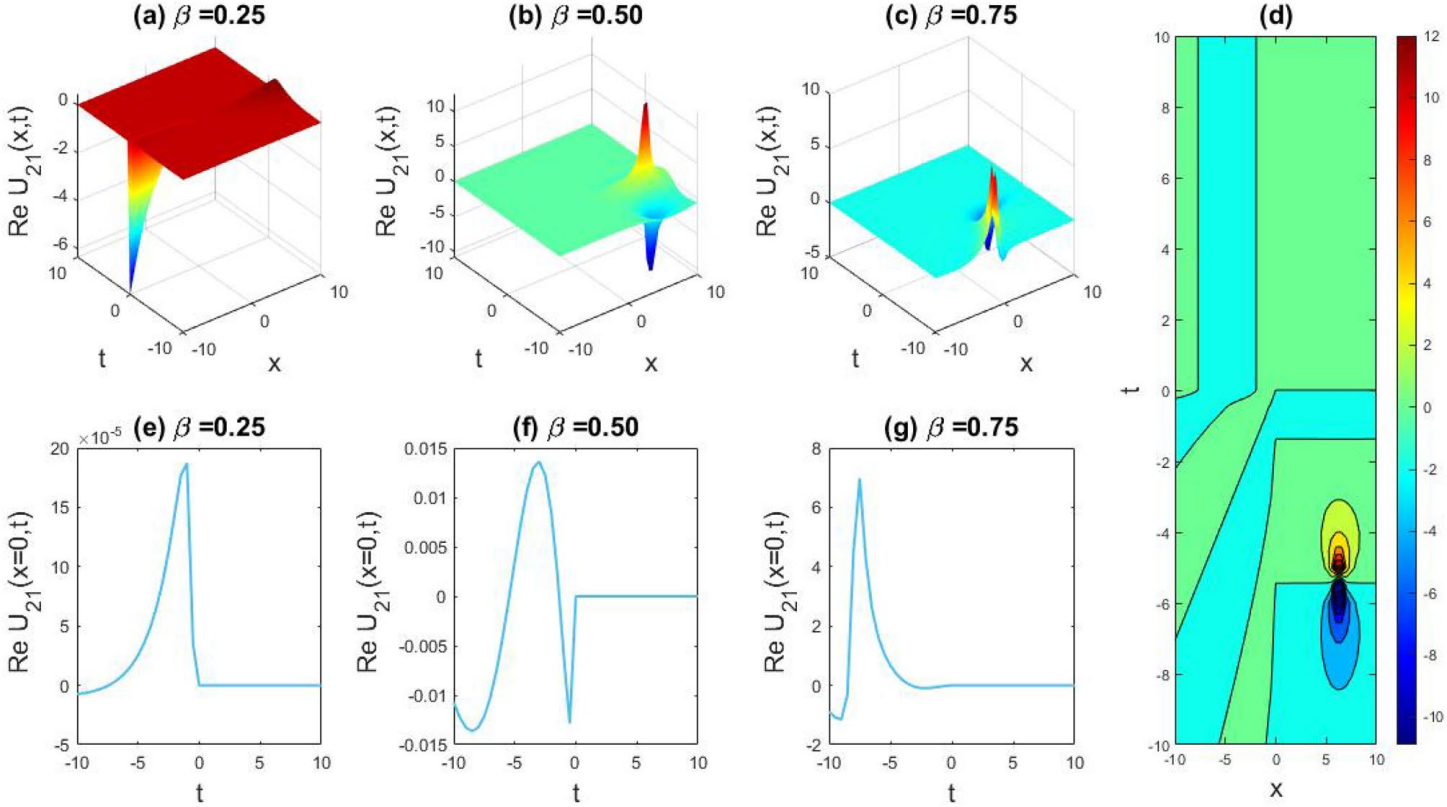
Exploring the dynamic behavior arising from the solution of ${U}_{21}(x, y, z, t)$ with parameters $A=-1, B=1, C=-1, b=-2, l=0.2, m=1, n=1.$ Subfigures (**a**–**c**) offer 3D perspectives, (**d**) presents the contour plot for $\beta =0.5,$ and (**e**–**g**) display 2D line graphs at $x=0$ corresponding to the associated 3D views.

**Figure 5:**
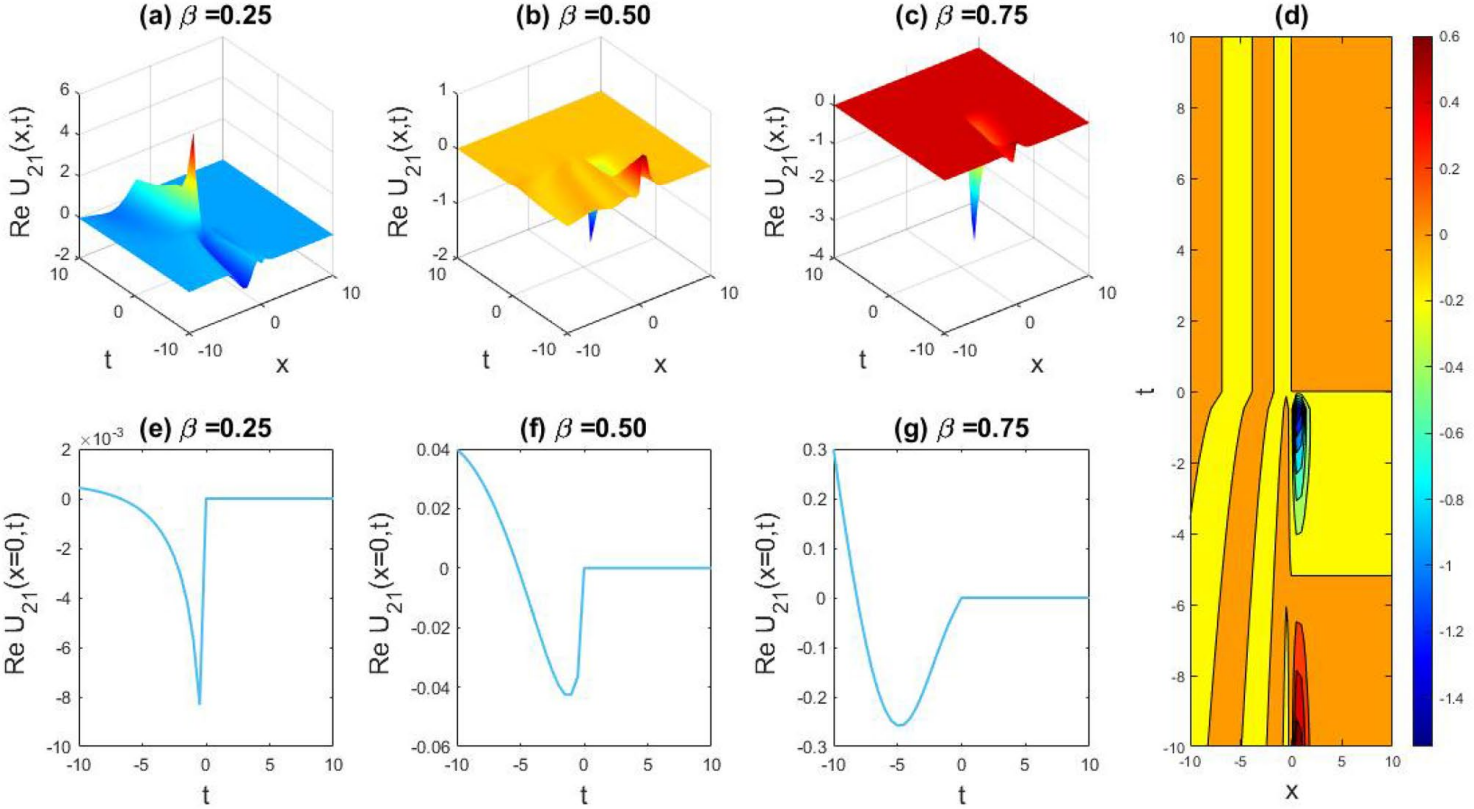
Capturing the dynamic patterns arising from the precise solution of ${U}_{21}(x, y, z, t)$ under the parameter set A = − 1, B = 1, C = − 1, b = − 1, l = 0.6, m = 1, n = 1. Subfigures (**a**–**c**) display 3D plot perspectives, with (**d**) featuring the contour plot for $\beta =0.5.$ Additionally, subfigures (**e**–**g**) depict 2D line graphs for $x=0$ corresponding to the respective 3D visualizations.

**Figure 6:**
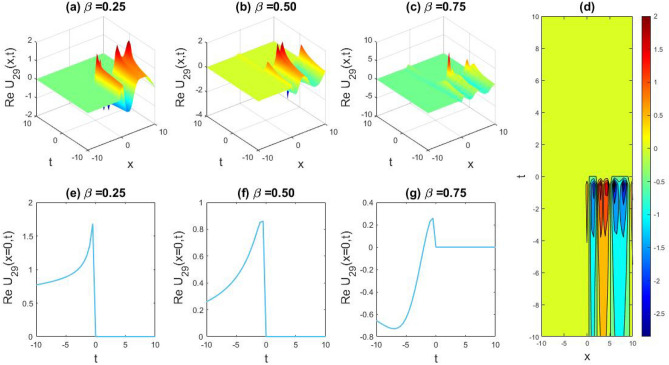
Unveiling the dynamical behavior of the solution of ${U}_{29}(x, y, z, t)$ with parameters $A=1, B=1, C=1, b=1, l=-1, m=1, n=1.$ Subfigures (**a**–**c**) showcase 3D plots, while (**d**) displays the contour plot for $\beta =0.5.$ Complementing this, subfigures (**e**–**g**) portray 2D line graphs at $x=0,$ corresponding to their respective 3D visualizations.

**Figure 7:**
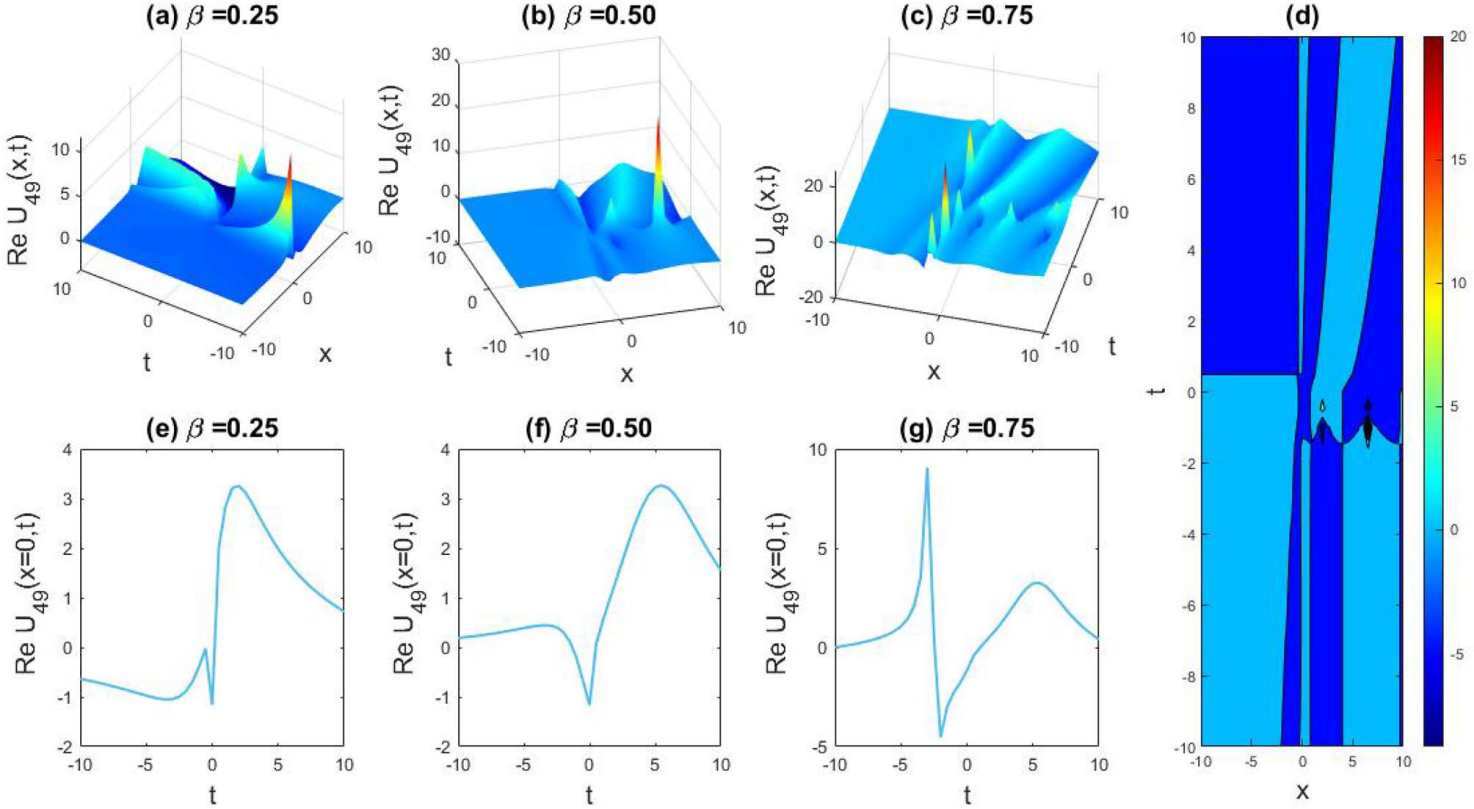
Illustrating the dynamic behavior derived from the solution of ${U}_{49}(x,y,z,t)$ with parameter values ${\text{A}}=2,\mathrm{ B}=2,\mathrm{ C}=-1,\mathrm{ b}=0.5,\mathrm{ l}=-1,\mathrm{ m}=1,\mathrm{ n}=1.$ Subfigures (**a**–**c**) provide 3D plot views, while (**d**) showcases the contour plot for $\beta =0.5$. Additionally, subfigures (**e**–**g**) feature 2D line graphs for $x=0$ corresponding to the respective figures (**a**–**c**).

**Figure 8:**
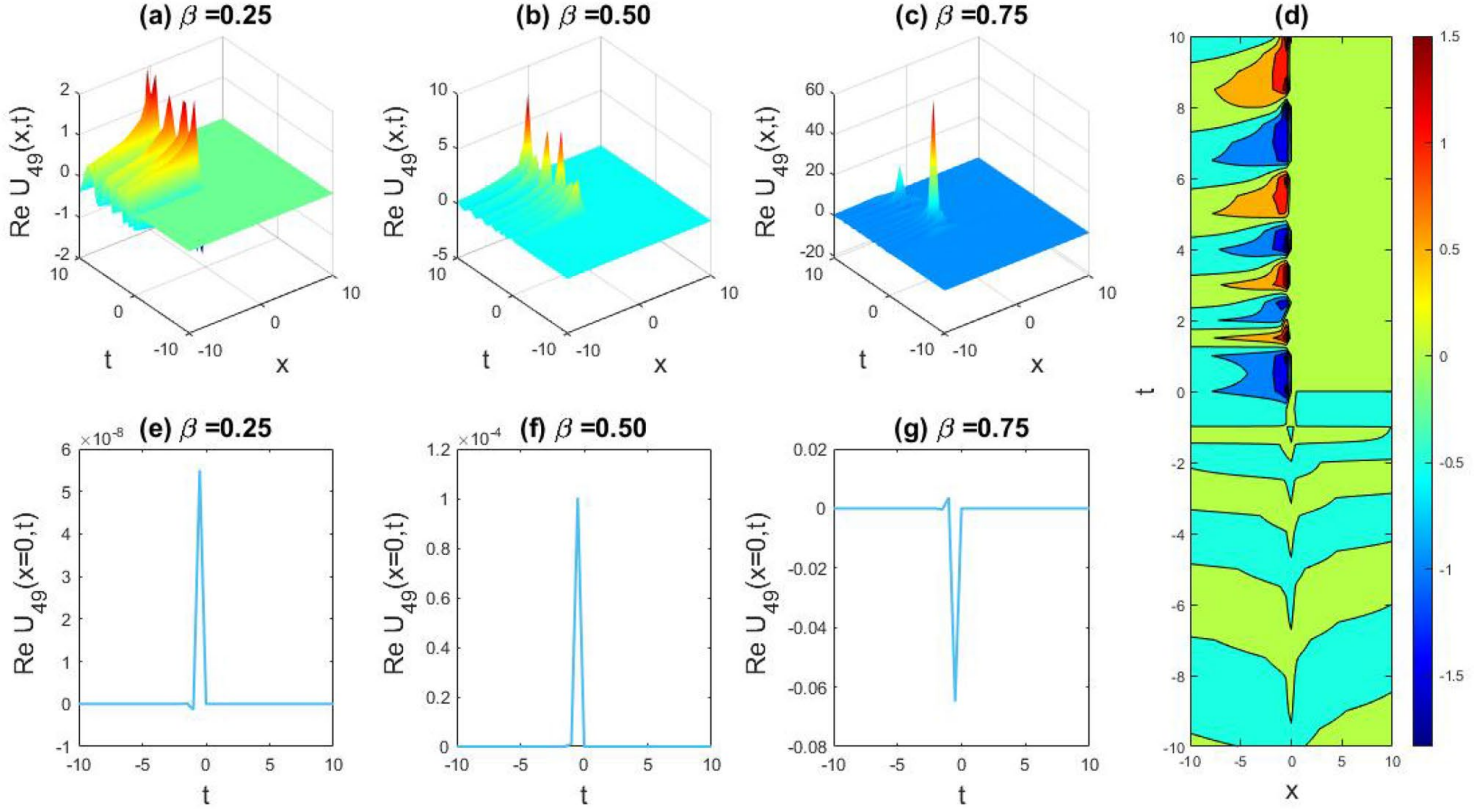
Capturing the dynamical evolution of ${U}_{49}(x, y, z, t$) with parameter values $A=2, B=2, C=-1, b=2, l=-0.2, m=1, n=1.$ Subfigures (**a**–**c**) showcase 3D plot views, (**d**) reveal the contour plot for $\beta =0.25,$ and subfigures (**e**–**g**) portray 2D line graphs for $x=0$ corresponding to (**a**–**c**) respectively.

**Figure 9:**
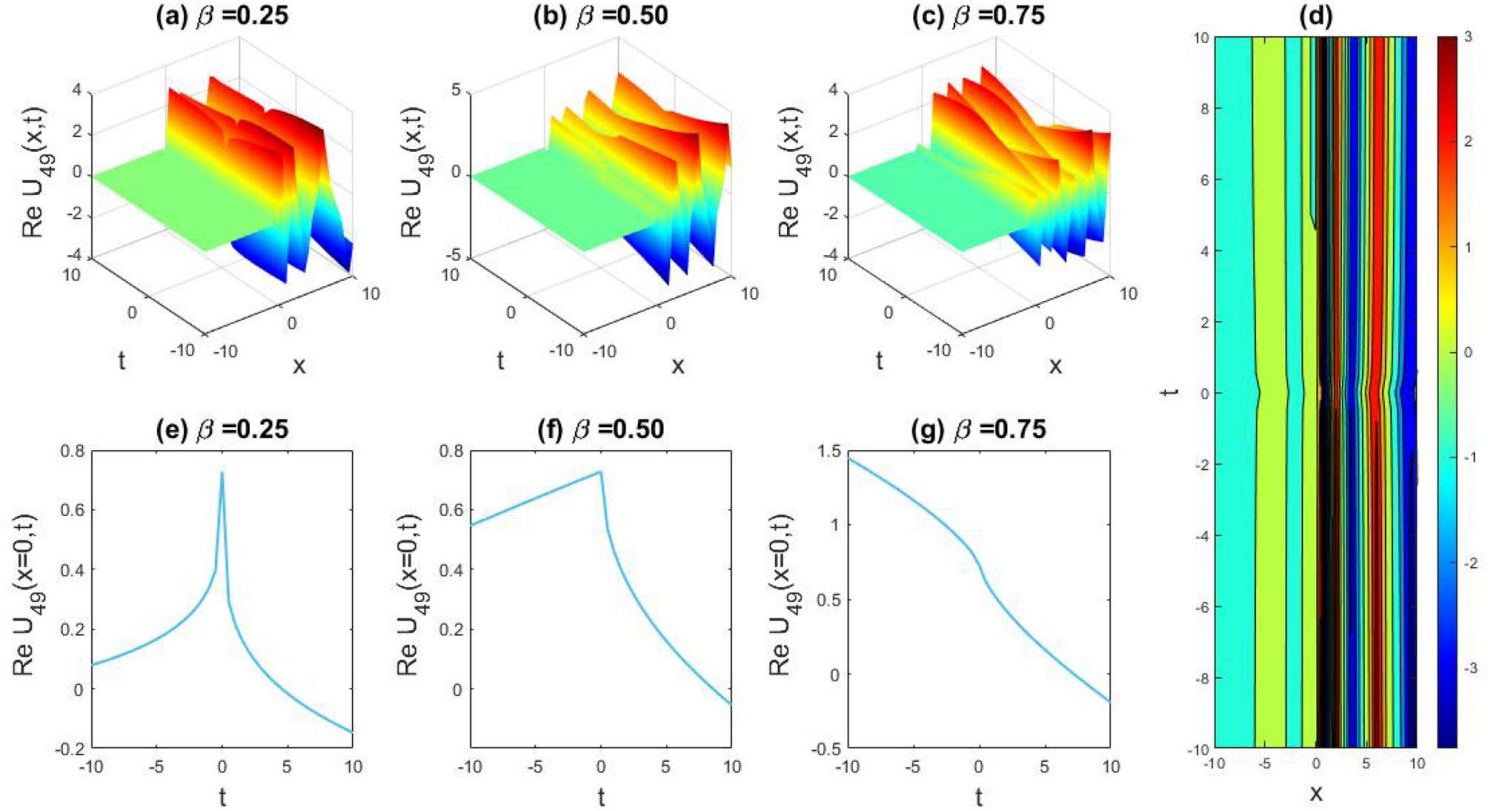
Capturing the dynamic behavior emerging from the solution of ${U}_{49}(x, y, z, t)$ with parameter values $A=2, B=2, C=-1, b=2, l=-1.5, m=1, n=1.$ Subfigures (**a**–**c**) showcase 3D plot views, (**d**) features the contour plot for $\beta =0.25,$ and subfigures (**e**–**g**) illustrate 2D line graphs for $x=0$ corresponding to figures (**a**–**c**) respectively.

**Figure 10:**
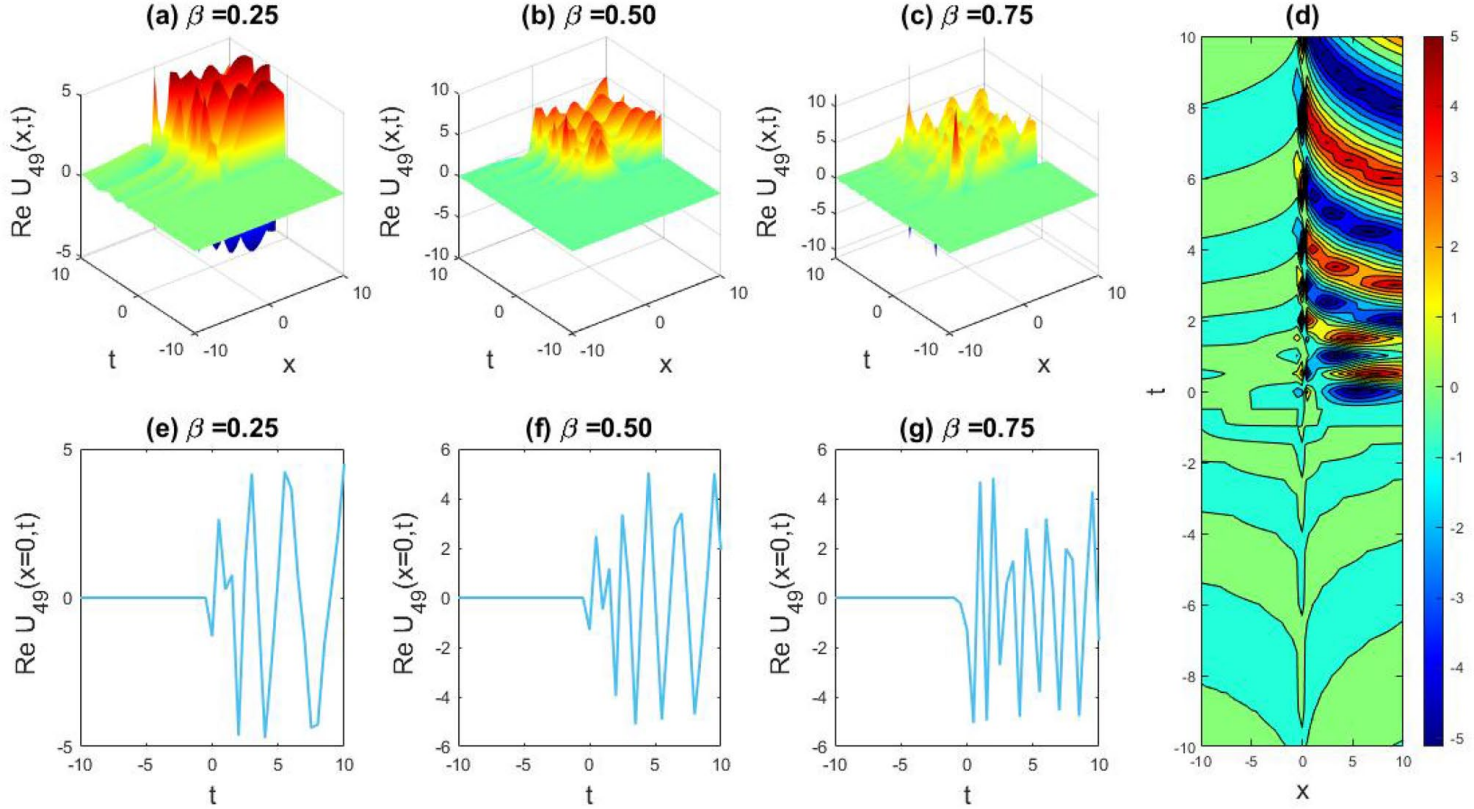
Exploring the dynamic behavior arising from the solution of ${U}_{49}(x, y, z, t)$ with parameters $A=2, B=2, C=-1, b=2, l=0.3, m=1, n=1.$ Subfigures (**a**–**c**) offer 3D perspectives, (**d**) present the contour plot for $\beta =0.25,$ and (**e**–**g**) display 2D line graphs at $x=0$ corresponding to the associated 3D views.

**Figure 11:**
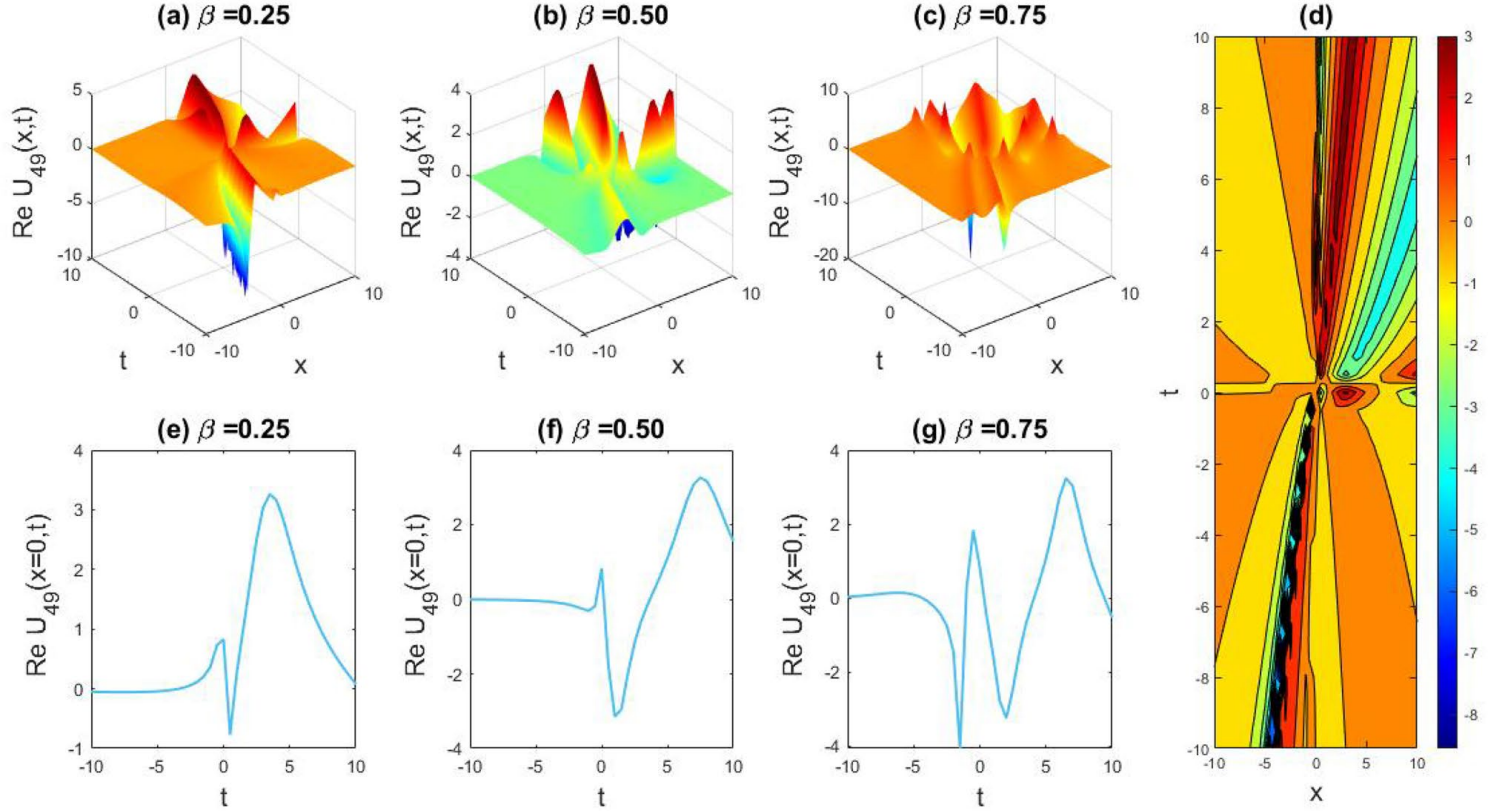
Capturing the dynamic patterns arising from the precise solution of ${U}_{49}(x, y, z, t)$ under the parameter set A = 2, B = 2, C = − 1, b = 2, l = − 0.5, m = 1, n = 1. Subfigures (**a**–**c**) display 3D plot perspectives, with (**d**) featuring the contour plot for $\beta =0.25.$ Additionally, subfigures (**e**–**g**) depict 2D line graphs for $x=0$ corresponding to the respective 3D visualizations.

The characteristics of fractionality are explored through various solutions of the fractional-order WBBM equations, as illustrated in Figs. 1, 2, 3, 4, 5, 6, 7, 8, 9, 10, 11, 12, providing a comprehensive perspective. For a clearer understanding, the fractionality characteristics are examined through several solutions of the WBBM equations of fractional order shown in Figs. 1, 2, 3, 4, 5, 6, 7, 8, 9, 10, 11, 12. In Fig. 1, we have presented the consequence of unrestricted variables and fractionality via 3D, 2D, as well as density plot assessment of dark soliton solution of equation ${U}_{9}(x,y,z,t)$ assuming the suitable parameters ${\text{A}}=-1,\mathrm{ B}=-1,\mathrm{ C}=-1,\mathrm{ b}=0.25,\mathrm{ l}=-0.2,\mathrm{ m}=1,\mathrm{ n}=1$. Figure 1a–c showcase 3D surface plots illustrating dark and bright soliton solutions corresponding to the parameters that are fractional $\beta =0.50$,$\beta =0.75$, and $\beta =0.90,$ correspondingly. Here, Fig. 1b portrays the dark soliton shape for the variation of parameter $\beta =0.50$ to $\beta =0.75,$ and when we altering the parameter $\beta =0.75 \; {\text{to}} \; \beta =0.90$ the dark soliton turns into the bright solution shape. Additionally, we noticed that the number of fluctuations of the bright and dark soliton solutions in Fig. 1e–g rises with the ascends of free parameters ${\text{l}},{\text{b}},\mathrm{ m}, \; \mathrm{and \; n}$. The graph of density for each group of figures is presented to facilitate a more comprehensive physical meaning. The line diagram in 2D illustrates both upper and lower frequencies, along with amplitudes, at various positions within the wave functional solutions presented in each section of the figure. According to the results mentioned earlier, it is essential to emphasize that nonlinearity factors with negative values $l$ and $b$, coupled with positive values of the dispersion factor in the model, can markedly impact the decrease or augmentation of profile intensity. In Fig. 2, we have shown a bright soliton solution of equation ${U}_{17}(x, y, z, t$) with parameter values $A=-1, B=-1, C=-1, b=0, l=-0.5, m=1, n=1$. Figure 2a–c depict the 3D plots of dark and bright soliton solutions considering the parameters $\beta =0.250$,$\beta =0.50$, and $\beta =0.75$, respectively. In this case, Fig. 2b depict the bright soliton shape for the alteration of parameter $\beta =0.250$ to $\beta =0.50,$ and when we alteration the parameter $\beta =0.50 \; {\text{to}} \; \beta =0.750$ the bright soliton turns into the singular form of a soliton. In Fig. 3, we showed the bright soliton solution of equation ${U}_{17}(x, y, z, t)$ with parameter values $A=-1, B=-1, C=-1, b=0, l=0.5, m=1, n=1$. On the other hand, Fig. 3a–c show the surface plots in 3D of dark and singular lump soliton solutions for the parameters $\beta =0.250$,$\beta =0.500$, and $\beta =0.750$ respectively. In this scenario, Fig. 3b depicts the bright soliton shape for the alteration of parameter $\beta =0.250$ to $\beta =0.500,$ and when we alteration the parameter $\beta =0.500 \; {\text{to}} \; \beta =0.75$ 0 the bright soliton transforms into the singular lump soliton shape In this scenario we altered the unrestricted parameters $l=0.5$ to $, l=-0.5$ from Fig. 2 in ${U}_{17}(x, y, z, t$) and its frequency distribution is depicted through a 2D plot in Fig. 3e–g. In Fig. 4, we showed the singular soliton solution of equation ${U}_{21}(x, y, z, t)$ with parameters $A=-1, B=1, C=-1, b=-2, l=0.2, m=1, n=1$. Figure 4a–c show the surface plots in 3D of singular soliton solution and lump solutions for the parameters $\beta =0.250$,$\beta =0.500$, and $\beta =0.750$ correspondingly. In this scenario, Fig. 4b depicts the singular form of soliton concerning the parameter change $\beta =0.250$ to $\beta =0.500$ and when we alteration the parameter $\beta =0.50 \; {\text{to}} \; \beta =0.75$ the singular soliton transforms into the lump soliton solution shape. In Fig. 5, we presented the singular soliton solution of equation ${U}_{21}(x, y, z, t)$ under the parameter set A = − 1, B = 1, C = − 1, b = − 1, l = 0.6, m = 1, n = 1. Figure 5a–c dipect the 3D designs of kink solution for the parameter $\beta =0.250$, $\beta =0.500$, and $\beta =0.750,$ correspondingly. In this scenario, Fig. 5b depicts the kink shape for the shift of parameter $\beta =0.25$ to $\beta =0.50$ and when we shift the parameter $\beta =0.500 \; {\text{to}} \; \beta =0.750$ the kink shape remains unchanged and only changed its original size and shape of its frequency. Here we altered the unrestricted parameters $l=0.2$ to $l=0.6$ from Fig. 4 in ${U}_{21}(x, y, z, t$) and its frequency distribution is depicted through a 2D plot in Fig. 5e–g. In Fig. 6, we showed the bright soliton solution of equation ${U}_{29}(x, y, z, t)$ with parameters $A=1, B=1, C=1, b=1, l=-1, m=1, n=1$. Figure 6a–c depict the 3D plots of bright soliton solutions for the parameters $\beta =0.250$,$\beta =0.500$, and $\beta =0.750$ respectively. In this case, Fig. 6b depicts the bright form of soliton for the shift of parameter $\beta =0.250$ to $\beta =0.500,$ and when we modify the parameter $\beta =0.500 \; {\text{to}} \; \beta =0.750$ the bright shape evolves into the dark form of soliton solution . In Fig. 7, we showed the bright soliton solution of equation ${U}_{49}(x,y,z,t)$ with parameter values ${\text{A}}=2,\mathrm{ B}=2,\mathrm{ C}=-1,\mathrm{ b}=0.5,\mathrm{ l}=-1,\mathrm{ m}=1,\mathrm{ n}=1$. Figure 7a–c depict the 3D surface diagram of soliton solutions for the values $\beta =0.250$,$\beta =0.500$, and $\beta =0.750$ respectively. In this case, Fig. 7b depicts the bright form of soliton for the variation of fractional value $\beta =0.250$ to $\beta =0.500$ and when we alter the value $\beta =0.500 \; {\text{to}} \; \beta =0.750$ the soliton shape remains unchanged. There is no dynamical variation for these parameters. In Fig. 8a–c, we found the multiple soliton solutions and two soliton solutions of equation ${U}_{49}(x, y, z, t$) with parameters values $A=2, B=2, C=-1, b=2, l=-0.2, m=1, n=1$ and bright multiple soliton shape for the equation ${U}_{49}(x, y, z, t)$ with the parameter values of $A=2, B=2, C=-1, b=2, l=-1.5, m=1, n=1$ depicted in Fig. 9a–c. Under this scenario, we changed the unrestricted parameters $l=-0.2$ to $l=1.5$ from Fig. 8 in ${U}_{49}(x, y, z, t$) and its frequency distribution is depicted through a 2D plot in Fig. 9e–g. In Fig. 10a–c, we obtained the periodic soliton solution shape of equation ${U}_{49}(x, y, z, t$) with parameter values $=2, B=2, C=-1, b=2, l=0.3, m=1, n=1.$ In Fig. 11a–c, we obtained bright soliton solution shape of equation ${U}_{49}(x, y, z, t$) with parameter values ${\text{A}}=2,\mathrm{ B}=2,\mathrm{ C}=-1,\mathrm{ b}=2,\mathrm{ l}=-0.5,\mathrm{ m}=1,\mathrm{ n}=1.$ Finally, in Fig. 12a–c, we obtained singular dark soliton solutions and two soliton solutions of equation ${U}_{53}(x, y, z, t)$ with the parameters $A=-2, B=2, C=1, b=0, l=-1.5, m=1, n=1$.

### Graphical representation

**Figure 12:**
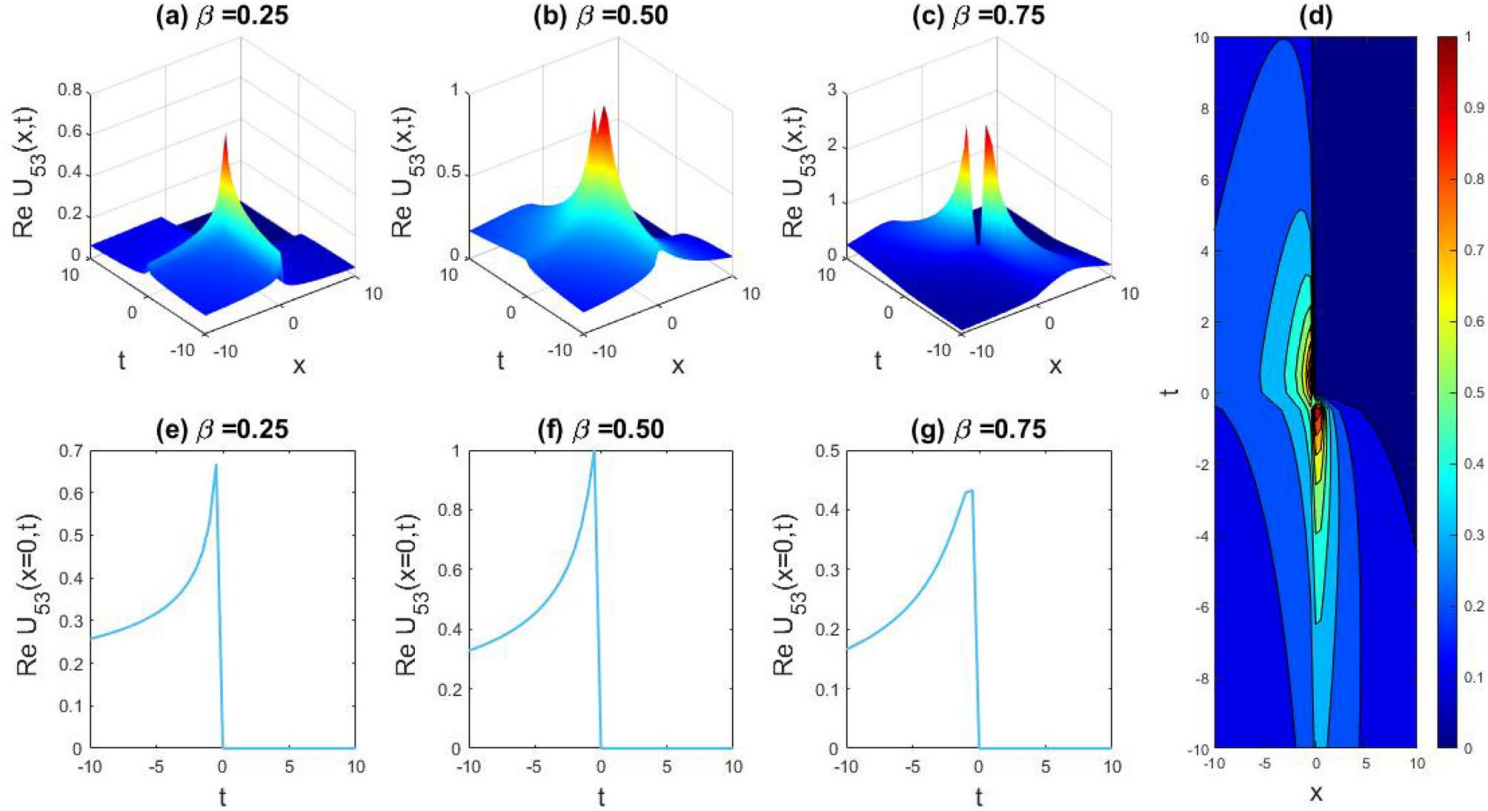
Unveiling the dynamical behavior of the solution of ${U}_{53}(x, y, z, t)$ with parameters $A=-2, B=2, C=1, b=0, l=-1.5, m=1, n=1.$ Subfigures (**a**–**c**) showcase 3D plots, while (**d**) displays the contour plot for $\beta =0.5.$ Complementing this, subfigures (**e**–**g**) portray 2D line graphs at $x=0,$ corresponding to their respective 3D visualizations.

## Conclusion

This work's main objective was to implement the expanded the Unified method to produce the new soliton solutions of space–time fractional 3D WBBM equation. Several new explicit solutions for hyperbolic, trigonometric, and rational functions were obtained as a result. These solutions can be expressed in various ways, such as lump solutions, dark-bright solitons, singular and multiple solitons, periodic, and other forms. Every solution that was found met the requirements of the matching equations. We display 2D and 3D contour graphs of a few of the found solutions. The study's findings might be convenient in illuminating the physical significance of some nonlinear models that emerge in the nonlinear sciences. To the best of our acquaintance, the received combined solitons have never been reported in other studies (Mamun et al.^2^; Shahen et al.^46^) in water wave mechanics. The significance of the derived results in many areas of physics, including met surfaces, nonlinear optical fibers, plasma physics, and metamaterials, makes it clear that they constitute a valuable addition to the literature already in existence.

## Data Availability

The datasets used and/or analysed during the current study available from the corresponding author on reasonable request.
